# Replicability of multivariate brain-behaviour associations depends on clinical profile

**DOI:** 10.1038/s42003-026-10364-z

**Published:** 2026-06-25

**Authors:** Michelle Wang, Brent C. McPherson, Bratislav Misic, Franco Pestilli, Celia M. T. Greenwood, Jean-Baptiste Poline

**Affiliations:** 1 McConnell Brain Imaging Centre, The Neuro (Montreal Neurological Institute-Hospital), McGill University, Montreal, QC Canada; 2 Department of Psychology, Department of Neuroscience, Center for Perceptual Systems, Center for Learning and Memory, The University of Texas, Austin, TX USA; 3 Department of Epidemiology, Biostatistics & Occupational Health, McGill University, Montreal, QC Canada; 4 Gerald Bronfman Department of Oncology, McGill University, Montreal, QC Canada; 5 Lady Davis Institute for Medical Research, Jewish General Hospital, Montreal, QC Canada

**Keywords:** Computational neuroscience, Communication and replication, Cognitive neuroscience

## Abstract

Recent work suggests that thousands of individuals are required in multivariate brain-behaviour analyses to obtain consistently replicable results. Some believe, however, that smaller sample sizes may be sufficient if specific subpopulations are targeted. We investigate how sample size and cohort composition influence the replicability of Canonical Correlation Analysis (CCA) results using the UK Biobank (*N* = 40,514). We apply CCA to diffusion-weighted magnetic resonance imaging (dMRI) phenotypes and cognitive assessment test scores. We define four participant cohorts based on clinical profile and find that, across all cohorts, sample sizes of ≈500 are needed to obtain replicable canonical correlations and variable loadings. The most targeted cohort (comprising individuals with a history of psychoactive substance use) requires much fewer samples to achieve similar or greater correlations than the other cohorts. Our findings support the idea that moderate sample sizes from targeted cohorts can be sufficient for obtaining replicable brain-behaviour associations.

## Abstract (web summary)

A study of 40,514 UK Biobank participants found that targeting a specific cohort based on clinical profile led to fewer samples being needed to achieve similar or greater correlations than the general population sample.

## Introduction

Traditional neuroimaging research has relied on small, locally acquired datasets that target specific scientific questions^1,2^: the median sample size of functional magnetic resonance imaging (fMRI) studies published in 2023 is estimated to be around 30^3^. This approach has resulted in unintentionally biased sampling of research participants and has played a substantial part in the current replication crisis, with many studies lacking the power to be generalizable to new samples^4,5^. Acquiring neuroimaging data can be time-consuming and expensive, and it is often not realistic to expect individual neuroscientists to collect hundreds of samples in order to answer a single research question. As a solution, the field has embraced large-scale efforts to acquire and share large, deeply phenotyped datasets that maximize the availability of quality research data for the largest number of research questions^6–8^, thus enabling the use of more complex models.

For magnetic resonance imaging (MRI) neuroimaging research, notable large and accessible datasets include studies from the UK Biobank (UKBB; ~40,000 participants)^9^, the Alzheimer’s Disease Neuroimaging Initiative (ADNI; ~2500 participants)^10^, the Human Connectome Project (HCP; ~1200 participants)^8^ and the Adolescent Brain and Cognitive Development study (ABCD; ~12,000 participants)^11^. These datasets include not only neuroimaging information, but also data from other modalities such as rich behavioural assessments, clinical data and genetic information. The breadth of information collected can enable powerful multivariate analyses. One such multivariate method is Canonical Correlation Analysis (CCA)^12^, which can be thought of as a correlation between two (or more) domains. If variables in a dataset can be separated by modality—for example, into brain and behaviour features—then CCA finds linear combinations of variables in each modality such that these linear combinations (latent factors called ‘canonical axes’) are maximally correlated with each other. The interpretation of the features that matter most in this model is a descriptive way of showing the relationship between these features; variables with a stronger contribution to the axis are weighted higher, having a stronger ‘loading’ onto the feature axis.

CCA has been used with samples like the HCP dataset to describe brain-behaviour relationships^13–15^. Unfortunately, CCA has notoriously poor replications to a single new sample^16,17^, meaning that variable loadings that are used to interpret the model can poorly reflect the true relationship of the underlying data. This limitation has recently been brought into the spotlight by Marek et al. (2022), who claimed that univariate and multivariate brain-wide association study (BWAS) methods like CCA need thousands of observations in order to accurately capture meaningful brain-behaviour relationships^18^. This has sparked many discussions in the neuroimaging community^19–37^, with some groups agreeing that such large sample sizes are indeed required^30,32^ and others arguing that smaller samples can be enough in some cases^23,24,26,31,36^.

Several of the responses to the Marek et al. (2022) study claim that more targeted samples (e.g. clinical populations) will create reproducible findings with only hundreds of samples due to the larger expected effect sizes in specific subpopulations^23,24,36^. This is an interesting distinction: most of the existing studies looking at how sample size impacts BWAS reproducibility used healthy populations with a large array of generic, non-specific features. Targeting more specific characteristics in a clinical sample may well improve the detection of relevant relationships, but this is yet untested.

In this study, we investigated the conjoint effects of sample size and sample composition on the reproducibility of multivariate models of brain-behaviour interactions in the UK Biobank (*N* = 40,514). Unlike most previous work related to the reproducibility of brain-behaviour associations^18,24,30,31,33,35^, we used structural brain measures derived from diffusion magnetic resonance imaging (dMRI) instead of functional connectivity or grey matter features. Features based on dMRI metrics are currently underreported for this kind of multivariate analysis, especially considering its emerging utility in tracking disease progression in different dementias^38,39^. Using dMRI phenotypes and cognitive assessment test scores, we attempted to replicate previous findings from Marek et al. describing the impact of sample size on the replicability of CCA results and investigated whether subsetting the full sample into different ‘cohorts’ influenced these results. In addition to comparing in-sample correlations with out-of-sample correlations, we also examined the set of variable contributions at different sample sizes. Overall, we find that CCA does not provide reliable results in samples with fewer than 500 individuals. However, we also show that individual clinical groups have a differing relationship than healthy controls. Our findings have important implications for smaller studies that are relying on more targeted populations to improve their effect sizes as an alternative to increasing sample size.

## Results

This study investigated the effect of cohort composition and sample size on brain-behaviour CCA reproducibility. We used data from 40,514 participants with brain imaging data from the UK Biobank dataset^9^. Brain data consisted of imaging-derived phenotypes (IDPs) from diffusion-weighted magnetic resonance imaging (dMRI). These included measures derived from diffusion tensor modelling (fractional anisotropy, FA; diffusion tensor mode, MO; and mean diffusivity, MD), as well as measures derived from Neurite Orientation Dispersion and Density Imaging (NODDI) (intracellular volume fraction, ICVF; isotropic or free water volume fraction, ISOVF; and orientation dispersion index, OD)^40^. The IDPs were obtained by computing the weighted mean of these dMRI measures over 27 white matter tracts^41^. Behavioural data consisted of key performance measures from nine cognitive function tests performed at the assessment centres. The tests targeted different domains of intelligence, including fluid intelligence/reasoning, matrix pattern completion, numeric memory, prospective memory, pairs matching, reaction time, symbol digit substitution, tower rearranging, and trail making. A total of 162 brain measures and 43 behavioural measures were included in this study.

From the Full cohort of 40,514 participants, we created three smaller cohorts targeting subsets of healthy participants (Healthy cohort, 6676 samples), participants with past psychoactive substance (e.g. alcohol, opioids, cannabinoids, sedatives or hypnotics, cocaine) use (Psychoactive cohort, 4725 samples), and participants with hypertension (Hypertension cohort, 7768 samples) (see ‘Methods’ for details). Demographic information for each cohort is shown in Supplementary Table 1.

Subsamples of each cohort were generated at up to 30 logarithmically spaced sample sizes, ranging from 50 to half of the total available data for each cohort. These subsamples were used to fit CCA models (Train set). For each model instance, a random half of the full cohort data (Test set) was used to assess the generalizability of the fitted CCA model on previously unseen data. We compared two types of CCA models: with and without cross-validation sampling. The model without cross-validation sampling consisted of a single CCA that was performed on the entire Train set, similar to many previous studies^13,15,18,31^. The model with cross-validation sampling was an ensemble model made up of 100 CCA instances, where each instance was fitted on a subset of the training data (see ‘Methods’ for details); this is similar to the ‘repeated k-fold cross-validation’ approach described in McPherson and Pestilli (2021)^14^, but has been adapted so that the model could be tested on fully held-out data. Each model was run 100 times for up to 30 sample sizes, yielding a total of up to 6000 fitted model instances for each cohort. For each fitted model instance, we obtained canonical correlations for the Train set and the Test set, as well as variable loadings (i.e. contributions) for each of the canonical axes for the Brain and Behaviour domains. Unless otherwise specified, results presented in this paper are for the first canonical axis (CA_1_).

### Replicable brain-behaviour CCA on the UK Biobank requires hundreds of samples

**Fig. 1:**
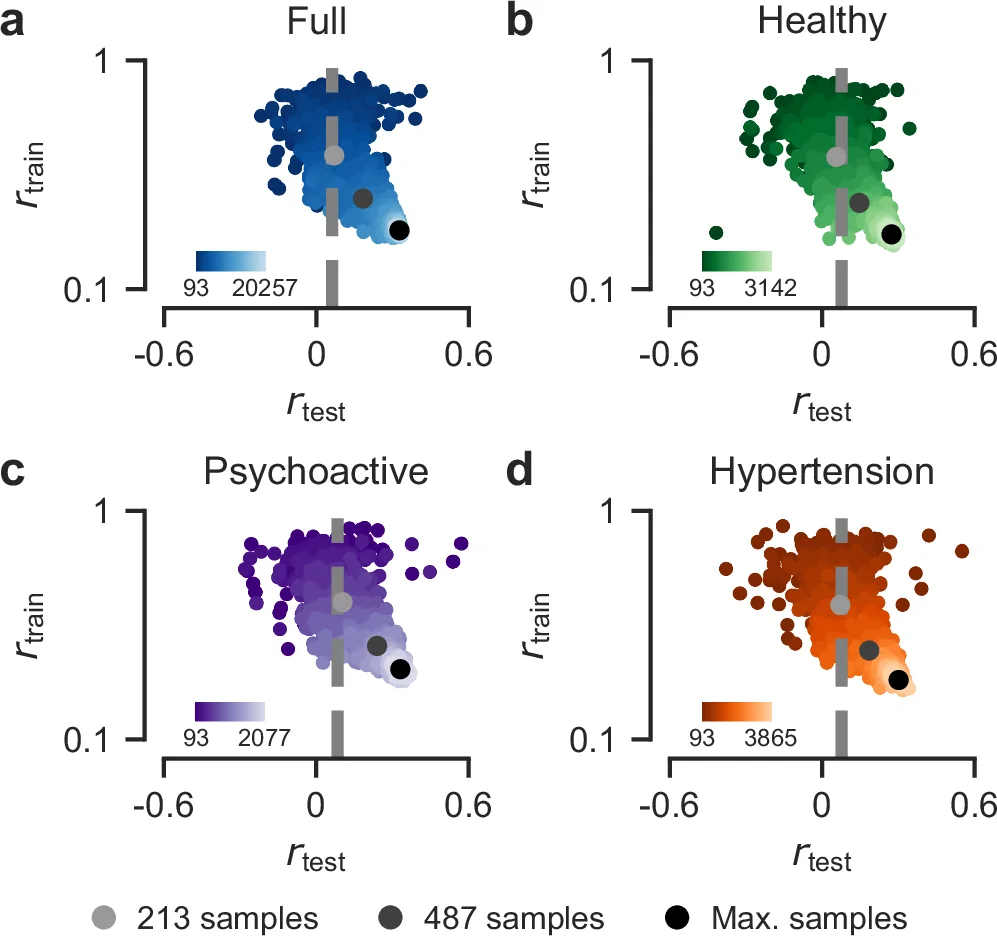
Train and test correlations across sample sizes and by cohort for the CCA model with cross-validation sampling. Train and test set correlations for each CCA model instance, coloured based on sample size. Grey dashed lines represent the 99th percentile of the null model distribution for the respective cohort (null models trained on largest available sample size). Larger dots represent mean ${r}_{{test}}$ at specific sample sizes (light grey: 213, dark grey: 487, black: maximum). For visualization purposes, only sample sizes of at least 93 are shown (see Supplementary Fig. 1 for all sample sizes). **a** Full cohort. **b** Healthy cohort. **c** Psychoactive cohort. **d** Hypertension cohort.

We investigated the reliability of brain-behaviour CCA at different sample sizes. As expected, for all four cohorts (Full, Healthy, Psychoactive, and Hypertension), mean canonical correlations for the Test set (${r}_{{test}}$) increase as the sample size increases, while those for the Train set (${r}_{{train}}$) decrease (Fig. 1). The mean ${r}_{{test}}$ at the largest sample size was 0.33 ± 0.01 (standard deviation) for the Full cohort, 0.27 ± 0.01 for the Healthy cohort, 0.33 ± 0.01 for the Psychoactive cohort, and 0.30 ± 0.01 for the Hypertension cohort. Across all cohorts, effect sizes were not statistically different from zero until at least a sample of *N* = 213 and required a sample of at least *N* = 487 to produce ${r}_{{test}}$ values that are significantly higher than those produced by null permutation models. This indicates that sample sizes of at least hundreds are generally needed to produce reliable results in such an analysis. Similar results were observed with the CCA model without cross-validation sampling (Supplementary Fig. 1).

### Correlation strength on unseen data differs between cohorts

**Fig. 2:**
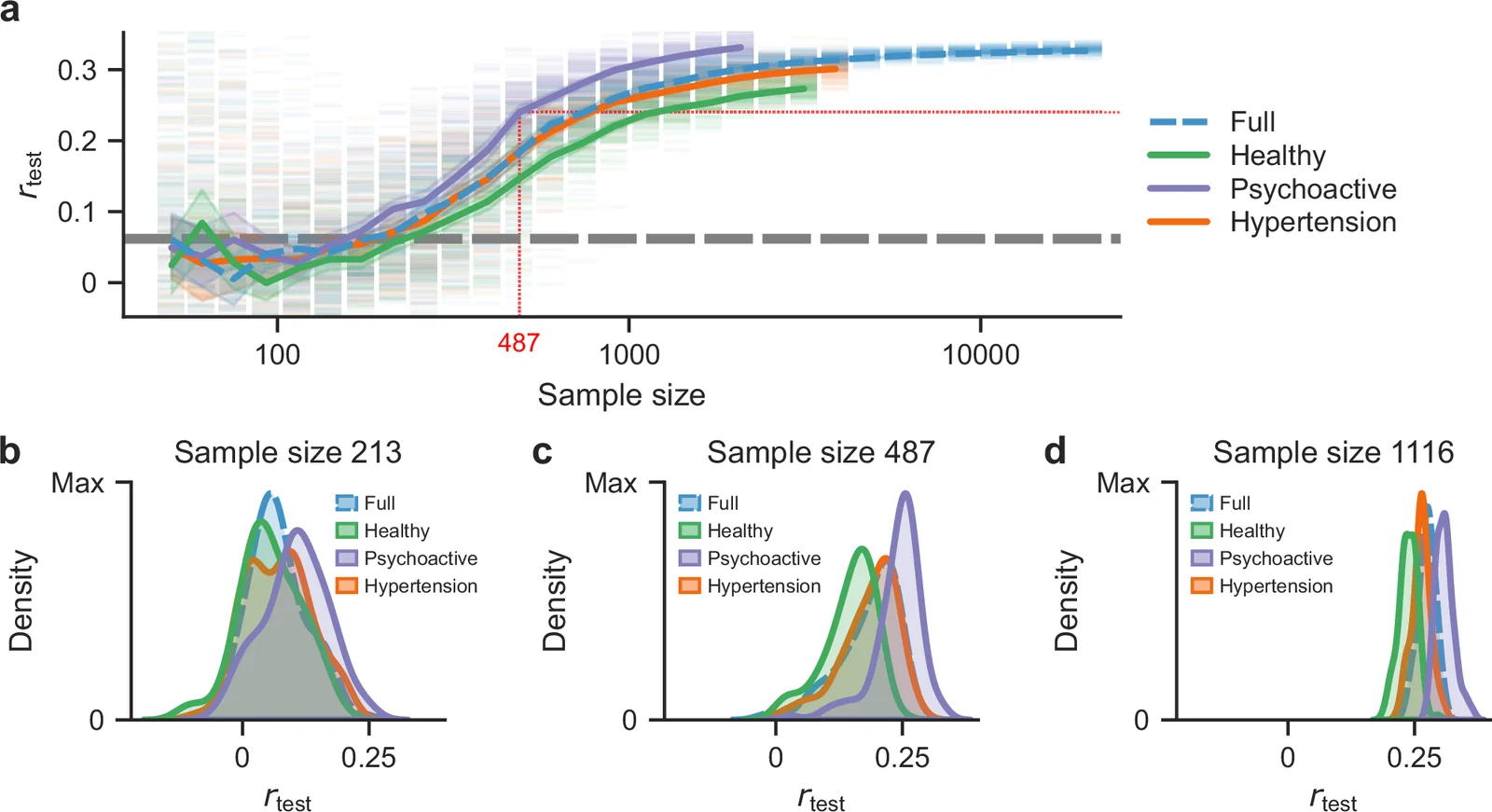
Replicability of canonical correlations across sample sizes and by cohort for the CCA model with cross-validation sampling. **a** Average test set correlation by sample size for the Full cohort and for each other cohort featured in this analysis. The grey dashed line represents the 99th percentile of the null model distribution for the Full cohort at its greatest sample size. Error bands denote 95% confidence intervals. **b**–**d** Kernel density estimation (KDE) distributions of the test set correlations for each cohort at sample sizes 213, 487, and 1116. The Psychoactive cohort consistently shows higher values compared to the other cohorts, while the Healthy cohort consistently shows lower values compared to the other cohorts.

Cohorts targeting different clinical conditions within the sample had a moderating effect on the brain-behaviour relationship across sample sizes. Although all four cohorts showed mean Test set correlations (${r}_{{test}}$) that increased with sample size, the corresponding increase to the correlations differed by cohort. Notably, the Psychoactive cohort consistently showed higher correlations compared to all other three cohorts (Fig. 2). At the sample size of 487, which corresponds to the lowest sample size for which there are sufficiently strong variable loadings for both Brain and Behavioural variables (as compared to null model loadings), the Psychoactive cohort had a mean ${r}_{{test}}$ of 0.24 ± 0.05, which is much higher than the mean ${r}_{{test}}$ values for the Healthy cohort (0.15 ± 0.05) (Fig. 2c). The Healthy cohort only reached a comparable ${r}_{{test}}$ (0.24 ± 0.02) with a sample size of 1116, which is more than twice as large as what was needed for the Psychoactive cohort. Mean ${r}_{{test}}$ values for the Hypertension and Full cohorts were close and largely remained in between the Healthy and Psychoactive cohort. Similar results were observed with the CCA model without cross-validation sampling (Supplementary Fig. 2).

To test the robustness of these results, we conducted an additional sensitivity analysis (Supplementary Fig. 3) where we varied the number of features used as input to the CCA model (see ‘Methods’). We compared the minimum sample size needed for each cohort to reach a mean ${r}_{{test}}$ of 0.24 (corresponding to the ${r}_{{test}}$ at sample size 487 for the Psychoactive cohort in Fig. 2). The Psychoactive cohort consistently required the lowest sample size (between 487 and 907) compared to the Full (between 907 and 1116), Healthy (between 1372 and 2077), and Hypertension (between 907 and 1372) cohorts (Supplementary Fig. 4).

### Variable loading order is preserved across sample sizes

**Fig. 3:**
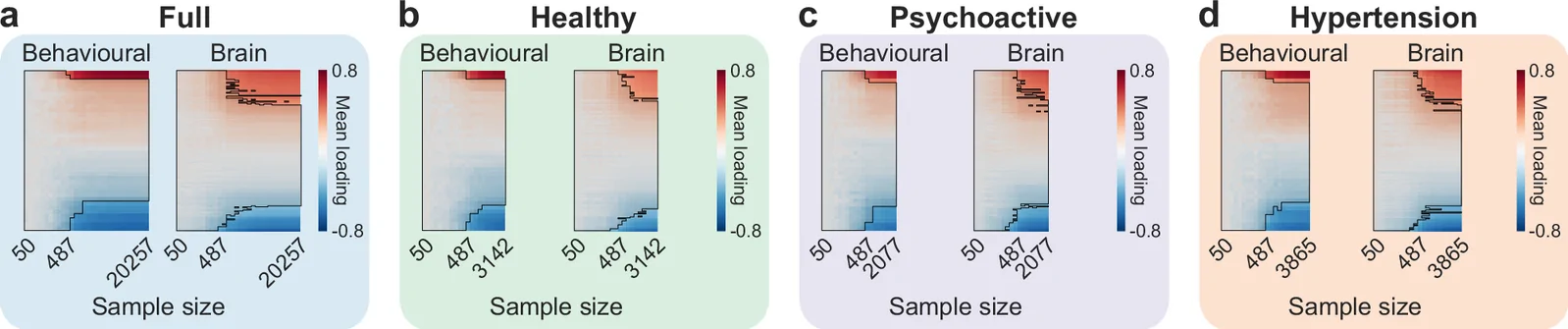
Mean loadings by sample size for brain and behavioural variables for each cohort for the CCA model with cross-validation sampling. Variable order is based on loading strengths at the largest sample size for each cohort. The saturation indicates the strength of the loading. Loadings whose mean value falls within the 2.5–97.5 null model percentile range are masked. **a** Full cohort. **b** Healthy cohort. **c** Psychoactive cohort. **d** Hypertension cohort.

We compared the average variable loadings across the repeated models at each sample size for the Brain and Behaviour domains (Fig. 3). For the Full cohort, the strongest positive and negative loadings (as determined by comparing with null model percentiles) are generally stable for sample sizes starting from around 500. This suggests that CCA with moderate sample sizes still provides a consistent estimation of the factor structure, even if the canonical correlations or factor loadings themselves are weaker. The variance of these estimates is relatively uniform for all variables at a given sample size and decreases as sample size increases (Supplementary Fig. 5). For sample sizes lower than 100–200, the variable loadings are unstable: there is no clear pattern in the loading order, and the loadings have lower means as well as higher variances. Hence, CCA models fitted on these smaller sample sizes should not be interpreted since they are not replicable. All four cohorts show a similar loading order pattern, with some loadings passing the null model threshold at even lower sample sizes (e.g. Brain loadings for the Psychoactive cohort at *N* = 262). Similar results were observed with the CCA model without cross-validation sampling (Supplementary Figs. 6 and 7).

Loadings that pass the null model threshold broadly agree between the four cohorts, though some minor differences exist (Supplementary Figs. 8–11). Specifically, brain features related to the ICVF fraction of thalamo-cortical connections were the strongest positive loadings, and the MD of the same thalamo-cortical connections were the weakest, consistent with established relationships of anisotropic and isotropic diffusion features^42–44^. In general, the strongest positive behavioural loadings were related to high task performance, especially for the symbol digit substitution task, while the reaction time and trail-making tasks consistently had the strongest negative behavioural loadings.

### Cross-validation sampling reduces effect size inflation, but only at low sample sizes

Without cross-validation sampling, CCA models trained on small samples show severe overfitting. For the Full cohort, at the smallest sample size of 50, the mean ${r}_{{train}}$ for the model without cross-validation sampling is 1.00 ± 0.00, while the mean ${r}_{{test}}$ is -0.03 ± 0.34. In contrast, for the model with cross-validation sampling, the mean ${r}_{{train}}$ and ${r}_{{test}}$ are 0.01 ± 0.24 and −0.01 ± 0.20, respectively. With larger sample sizes, the two CCA models produce much closer results for both ${r}_{{train}}$ and ${r}_{{test}}$ (Supplementary Fig. 12). We observe similar results for all four cohorts.

**Fig. 4:**
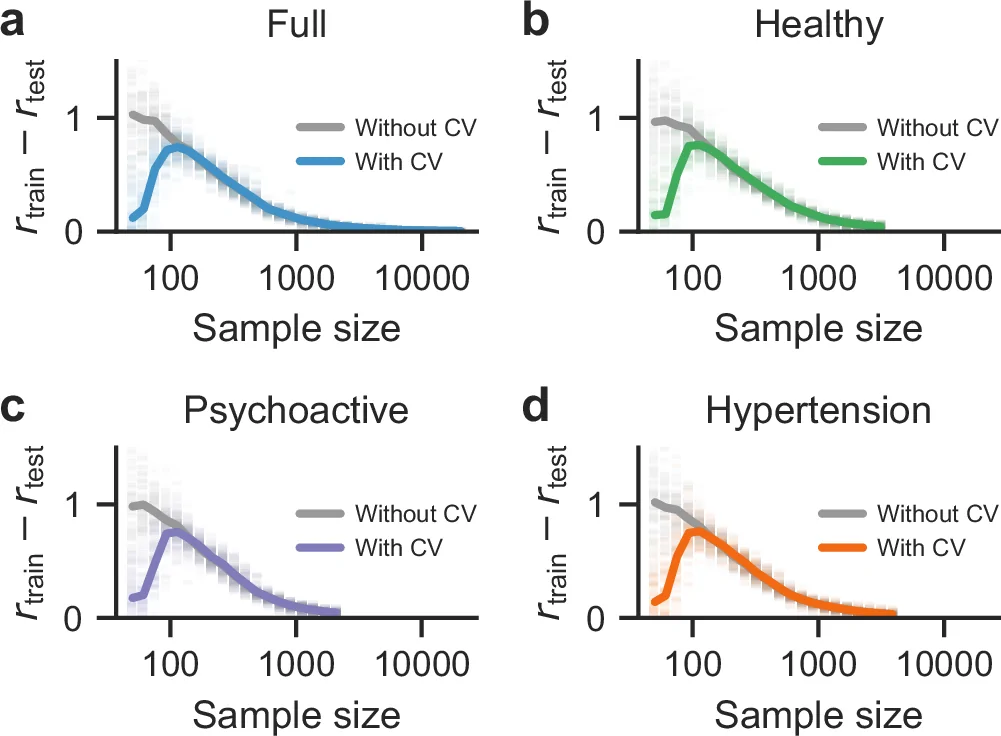
Comparison of effect size inflation between the two types of models for each cohort. Full lines: CCA model with cross-validation sampling. Dashed lines: CCA model without cross-validation sampling. Error bands denote 95% confidence intervals. **a** Full cohort. **b** Healthy cohort. **c** Psychoactive cohort. **d** Hypertension cohort.

When it comes to the effect size inflation, as measured by the difference between Train set and Test set correlations, we find that CCA with cross-validation sampling reduces effect size inflation compared to CCA without cross-validation sampling, though only for sample sizes up to around 100 (Fig. 4). This finding is not unexpected since a larger Train set already reduces chances of overfitting. However, much larger sample sizes (greater than *N* = 1000) are needed to obtain an inflation value that is close to zero. Cross-validated and non-cross-validated models both have the same levels of overfitting of sample sizes over sample sizes of 100.

## Discussion

In this study, we investigated the impact of cohort composition and sample size on the replicability of multivariate brain-behaviour relationships obtained from CCA methods. Utilizing data from the UK Biobank, we created training and test samples across a range of sample sizes from 50 to 20,257 for the full population and targeted clinical cohorts. We consistently reproduce general trends from Marek et al. 2022 showing that out-of-sample correlation increases as sample size increases. Sample sizes in the low hundreds or below led to non-replicable CCA model results and unstable variable loadings. However, although the largest sample sizes produce the best results, we find that sample sizes close to 500 are sufficient to obtain reproducible correlations and meaningful variable loadings with our specific study data. Moreover, variable loadings are stable even for these lower sample sizes (*N* ≈ 500), and their order is preserved as sample size increases, indicating that they are reliably detecting a coherent underlying relationship of the variables. Our findings are therefore in line with several recent articles arguing that moderate sample sizes—in the order of hundreds, not thousands—may be enough for reproducible brain-behaviour associations^24,31^.

We found that using a targeted subset of participants with current or past psychoactive substance use resulted in higher replicability of brain-behaviour canonical correlations when compared to other participant cohorts at the same sample size. Psychoactive substance use is known to affect both cognitive function^45^ and white matter microstructure^46^: a change in either domain could change the observed relationship in our cohort^47^. The exact cause of this effect is unclear from our current analysis, and a deeper interpretation of this specific finding was beyond the scope of our study. However, sensitivity analyses suggest that the behavioural domain could be important, as the effect is less marked with fewer behavioural measures (Supplementary Fig. 3), but more systematic analyses would be needed to draw concrete conclusions.

Our main findings are consistent with the claim that lower sample sizes may be sufficient when effect sizes are expected to be larger due to study design^23,24,26,33,36^. It should be noted that our study only focused on varying cohort composition, while other factors, such as feature selection, likely also impact brain-behaviour associations. For example, collecting a more specialized set of features could lead to a sufficiently powered CCA with a much smaller sample. The same could be said about MRI modality, though a recent review of multivariate brain-behaviour relationship comparisons across clinical conditions found that cortical morphology and white matter association tracts had an overall stronger impact on a brain-behaviour axis than resting state fMRI^47^. In parallel, analytical choices such as using regularized CCA^48^ or other models (e.g. Bayesian methods) could impact the replicability of results as well. Such comparisons were beyond the scope of this study, but could form the basis of future work in this field.

We tested CCA models with and without cross-validation sampling (implemented as an ensemble model of 100 individual CCA models), and found that cross-validation sampling reduced the amount of overfitting on the training data, but only for very low sample sizes of around 100 or less. Even the largest sample size in this range was not enough to produce reliable brain-behaviour relationships in our other analyses. Nevertheless, using cross-validation sampling on small datasets can inform on sample size adequacy and give a more realistic expectation of model performance on previously unseen data.

In general, although our results support the notion that targeted cohort selection can reduce the sample size requirements for multivariate brain-behaviour analyses, we acknowledge that this may not always be the case depending on the cohort. Cohort specification is a procedure that can span multiple dimensions of subject characteristics: disease itself is a multidimensional trait, and it typically interacts with demographic, socioeconomic, and genetic features. It is likely that not all dimensions would impact brain-behaviour associations in the same manner. Therefore, in some cases, it is possible that what appears to be a more clinically homogeneous cohort shows in practice smaller brain-behaviour effect sizes.

Overall, we believe this study answers some of the concerns regarding CCA analyses by directly testing the impact of sample size and composition on real data. We hope these findings can inform future studies when it comes to experimental design and data acquisition planning. In addition, we invite the scientific community to begin a discussion on the interpretation and downstream applications of CCA results. Specifically, what determines a significant model in the context of CCA has been poorly defined in previous work. In some cases, only results for the first canonical axis are presented^18,32^, although other axes may also produce significant canonical correlations. Moreover, the current literature seems to show little consideration for the reliability of feature contributions to canonical loadings (with some exceptions^32,35,48^). For example, in our study, we found that the first sample size with a significant first canonical axis was in fact lower than 487 for most cohorts; however, these canonical axes did not have strong loadings in both the Brain and Behavioural domains at these sample sizes. Would it be accurate to interpret these smaller sample sizes as adequate for capturing a multivariate comparison when none of the features provides statistically non-zero variable loadings? The statistical reliability of parameter estimates may be less relevant when the only interest is the descriptive ability of the canonical correlation between modalities. However, even in this context, often the strongest loading features of the axes are used to interpret the composition of the respective domains. A statistically derived threshold for parameters would allow for these and similar multivariate models to serve as testable, predictive models instead of purely descriptive.

To conclude, this study replicates some of the results found in Marek et al. (2022). Specifically, we find that out-of-sample CCA effect sizes are low at the sample sizes featured in most neuroimaging studies (≪500). Our findings complement previous work in the literature by showing that cohort composition can play a substantial role on the magnitude of these out-of-sample effect sizes. Further, when we looked beyond effect sizes, we found that we were able to obtain stable variable loadings at sample sizes of around 500. Overall, we find that reliable and meaningful CCA models still need large datasets, but with sample sizes on the order of hundreds, not thousands.

## Methods

### Dataset

This study used structural brain and behavioural measures from the UK Biobank (https://www.ukbiobank.ac.uk/; Application 45551). The UK Biobank project received approval from the National Information Governance Board for Health and Social Care and the National Health Service North West Centre for Research Ethics Committee (reference 11/NW/0382). Informed consent was obtained from all participants, and all ethical regulations relevant to human research participants were followed.

We extracted dMRI measures and cognitive test results to form a Brain dataset and a Behavioural dataset, respectively. Demographic, physical and physiological variables were treated as confounds and regressed out of the Brain and Behavioural datasets. Participants with completely missing data in any of the datasets were excluded from the entire analysis. Our final full dataset consisted of 40,514 participants.

UK Biobank participants typically underwent multiple visits (called ‘instances’) to the assessment centres. Unless otherwise specified in this text, data used in this study are from the first imaging visit (Instance 2). Data from subsequent imaging visits were not included because longitudinal features were beyond the scope of this analysis. The UK Biobank data showcase website (https://biobank.ndph.ox.ac.uk/showcase/) was used to identify relevant categories and fields for the three datasets described below. Only numerical (integer, categorical, or continuous) data fields were considered.

### Brain variables

Structural brain variables consisted of pre-computed phenotypes derived from dMRI scans. We included a subset of the fields from Category 135 (‘dMRI weighted means’), namely the weighted-mean measures for fractional anisotropy (FA), diffusion tensor mode (MO), mean diffusivity (MD), intracellular volume fraction (ICVF), isotropic or free water volume fraction (ISOVF), and orientation dispersion index (OD). These values are derived from fitted diffusion tensor and neurite orientation and dispersion density index (NODDI) model parameter values and were averaged within a set of 27 white matter tract regions. The dMRI acquisition methods and processing pipeline have been previously described by the UK Biobank neuroimaging analysis group^41^. A total of 162 structural brain variables were selected.

### Behavioural variables

A set of 43 cognitive function measures were selected for the Behavioural dataset. These measures consisted of results from a series of nine cognitive tests performed at the assessment centres: reaction time, numeric memory, fluid intelligence/reasoning, trail making, matrix pattern completion, tower rearranging, symbol digit substitution, prospective memory, and pairs matching (Category 100026: ‘Cognitive function’). For 15 measures, participants’ responses were binarized to indicate whether the participant answered correctly or not; these consisted of the fluid intelligence test items (Fields 4924, 4935, 4946, 4957, 4968, 4979, 4990, 5001, 5012, 5556, 5699, 5779, 5790, 5866) and Field 20018 ‘Prospective memory result’, where only participants who provided the right answer in their first attempt were considered correct. For cognitive assessments that involved multiple rounds (reaction time, numeric memory, trail making, matrix pattern completion, and pairs matching; Fields 398, 399, 400, 403, 404, 4255, 6333, 6770, 6771, 6772, 6773), individual round data were aggregated into summary measures of mean and standard deviation.

### Confounding variables

18 confounding variables were identified and regressed out of the Brain and Behavioural datasets. These measures were chosen based on methods described in previous work^13,14^. They consisted of the following:

1. Primary demographics, including sex and assessment centre but excluding age (Category 1001, except Field 21003 ‘Age when attended assessment centre’ and Field 34 ‘Year of birth’);
2. Blood pressure (Category 100011, except for Field 96 ‘Time since interview start at which blood pressure screen(s) shown’);
3. Handedness (Field 1707);
4. Pulse rate (Field 4194);
5. Standing height (Field 50);
6. Weight (Field 21002) and weight method (Field 21).

Some demographic fields, such as sex, were not recorded during the first imaging visit; in these cases, the value at Instance 0 was used for all participants.

Age-related data were not included as confound variables due to evidence that age may be more of an effect modifier than a confounder: brain-behaviour CCA associations are strongly linked with age, and including age as a confound reduces canonical correlations to near zero^14^.

### Cohort selection

Cohorts were formed based on the International Classification of Diseases 10 (ICD-10) summary diagnosis codes available for each participant (Fields 41270, 41202, 41204, and 41201). The selection criteria consisted of the following:

1. *Full:* all available participants (i.e. no filtering);
2. *Healthy:* participants without any diagnosis code;
3. *Psychoactive:* participants with code Z86.4 ‘Personal history of psychoactive substance abuse’ or any code in the F10-F19 categories (‘Mental and behavioural disorders due to psychoactive substance use’) (see https://icd.who.int/browse10/2019/en#/F10-F19 for more detailed information);
4. *Hypertension:* participants with code I10 ‘Essential (primary) hypertension’.

The final cohort sizes were the following: 40,514 for Full, 6676 for Healthy, 4725 for Psychoactive, and 7768 for Hypertension. By definition, the Full cohort contained all the participants in the other cohorts (as well as additional ones), and the Healthy cohort had no overlap with either the Psychoactive or the Hypertension cohort. Summary demographic information for each of the four cohorts is available in Supplementary Table 1. The Psychoactive and Hypertension cohorts had 1989 participants in common – these did not seem to be driving the effects we found, as removing them from the analysis still showed that the Psychoactive cohort needed less samples compared to the Healthy or Hypertension cohorts (Supplementary Fig. 13).

### Data preprocessing

All categorical variables were one-hot encoded. Variables were removed if they had more than 50% missing data or if more than 80% of participants had the same value. The 80% threshold for low variability was chosen to ensure the impossibility of obtaining data with no variability at any point during the repeated five-fold cross-validation procedure.

Preprocessing steps for confounding variables consisted of iterative imputation, inverse normal transformation and *z*-score standardization using the median and interquartile range. Squared versions of integer or continuous demographic variables were also added to account for some nonlinear effects when deconfounding.

Identical preprocessing pipelines were used for each of the Brain and Behavioural datasets. Variables were first normalized and *z*-scored (ignoring missing values). Then, a linear regression model was used to regress out the preprocessed confounding variables from the data. Principal Component Analysis (PCA) was then applied to reduce the dimension of the two datasets to 20 principal components (PCs) each. PCA was performed in a way that did not require imputing missing values: the data covariance matrix was first computed while ignoring missing data, then this (possibly non-diagonalizable) covariance matrix was projected to the nearest symmetric positive definite matrix, which was used in the eigenvector decomposition^13,14^.

### Canonical correlation analysis

CCA consists of solving the following optimization problem:

1 $${\max}_{{{w}_{1},\,{w}_{2}}} \left({{\mathrm{correlation}}}\left({D}_{1}{w}_{1},\,{D}_{2}{w}_{2}\right)\right)$$

where ${D}_{1}$ and ${D}_{2}$ are two datasets (with observations as rows and features as columns), and ${w}_{1}$ and ${w}_{2}$ are weights that linearly combine columns from these datasets into *canonical factors* (or *canonical variables*) that are maximally correlated with each other. Additional pairs of canonical factors can be obtained by solving the same optimization problem, under the constraint that a pair of canonical factors cannot be correlated with another pair of canonical factors (i.e. they must be orthogonal). CCA can also be seen as finding a latent space such that projections of ${D}_{1}$ and ${D}_{2}$ onto that space have the highest correlation in the first latent dimension (first canonical axis, CA_1_), the second-highest correlation in the second latent dimension (CA_2_), and so on for all CAs.

In addition to the previously described canonical factors, the main results of an analysis involving CCA also include *canonical correlations* and *canonical loadings*. Canonical correlations are the correlations between each pair of canonical factors, and canonical loadings describe how features from the original data contribute to the canonical factors. Canonical loadings are obtained by correlating the original data matrices with the canonical factors.

### General data sampling and model fitting procedure

Data were split into Train and Test sets. The Train set was used for model fitting, including any cross-validation/ensembling. The Test set was used for model evaluation and always matched the maximum Train set size for each cohort (20,257 for the Full cohort, 3142 for the Healthy cohort, 2077 for the Psychoactive cohort, and 3865 for the Hypertension cohort). For each sample size, we generated a Train set by sampling (without replacement) from the participants who were not in the Test set. Sample sizes ranged from 50 to 20,257 (half of the total available data) and were chosen to be evenly spaced on a log scale. The exact sample sizes used were 50, 61, 75, 93, 114, 140, 173, 213, 262, 322, 396, 487, 599, 737, 907, 1116, 1372, 1688, 2077, 2555, 3142, 3865, 4755, 5848, 7194, 8849, 10884, 13388, 16468, and 20257. This process (including the initial 50% Test set sampling) was repeated 100 times for each cohort and each CCA model. All 30 sample sizes were used for the Full cohort; the first 21 sample sizes (up to 3142) were used for the Healthy cohort, the first 22 sample sizes (up to 3865) were used for the Hypertension cohort, and the first 19 sample sizes (up to 2077) were used for the Psychoactive cohort. Within a cohort, the same samples were used for the CCA model without cross-validation and the CCA model with repeated cross-validation.

To ensure that there was no data leakage between Train sets and Test sets, the parameters used in the preprocessing pipelines and the CCA model were estimated using data from the Train set only. Similarly, feature selection was done using only data from the Train set. Since each model fit was repeated 100 times on different Train sets, this resulted in potentially different numbers of features for the same model trained on different data, especially for smaller sample sizes.

### CCA without cross-validation sampling

We used the basic (non-regularized) CCA implementation from the CCA-Zoo Python package^49^ (version 1.10.11), which uses eigenvector decomposition to find the optimal transformation weights for the two datasets. The number of CAs was set to 20, which was the dimension of the Brain and Behavioural datasets after dimensionality reduction with PCA. This approach did not use cross-validation and consisted of training a single instance of the model on the entire Train set.

### CCA with cross-validation sampling

The cross-validation sampling approach was modified from the method described in a previous study by McPherson and Pestili^14^. Our approach consists of an ensemble model procedure where five-fold cross-validation is repeated 20 times, yielding a total of 100 models. We then applied each fitted model to the full Train or Test set to obtain 100 sets of canonical factors. To account for arbitrary changes in direction that could have occurred between the 100 models, the canonical factors were aligned to the canonical factors from the CCA model without cross-validation sampling using the orthogonal Procrustes method^50–52^, using rotation matrices derived from the Train set canonical factors. The aligned canonical factors were then averaged to produce a single set of canonical factors for the overall (ensemble) model. Canonical correlations for the Train and Test sets were computed using the averaged canonical factors. Canonical loadings were obtained by correlating the normalized, z-scored and deconfounded data matrices (averaged from the 100 models) with the averaged canonical factors from the ensemble model.

### Null models

The null models were fitted in exactly the same way as the regular models, except that the Brain and Behavioural datasets were shuffled so that the participant order between them would not match. The maximum sample size was used for each of the four cohorts. Each null model was run 1000 times.

### Statistics and reproducibility

All analyses were performed using the Python programming language. The main packages used to implement the CCA models were scikit-learn^53^ and CCA-Zoo^49,54^. Other packages included pandas^55,56^ and NumPy^57^ for data wrangling, as well as Matplotlib^58,59^ and seaborn^60^ for plotting.

CCA models were run 100 times for each combination of sample size, cohort and model type (with or without cross-validation sampling). Null models were run 1000 times. Strong canonical loadings were defined as those whose mean values fell outside the 2.5–97.5 percentile range of the null model distributions.

### Reporting summary

Further information on research design is available in the Nature Portfolio Reporting Summary linked to this article.

## Supplementary information

Supplementary Information

Reporting Summary

Transparent Peer Review file

## Data Availability

Data used in this study can be obtained by applying for access to the UK Biobank database (https://www.ukbiobank.ac.uk/). Source data for figures is available at https://github.com/neurodatascience/ukbb-cca and has been deposited to the Zenodo data repository^61^.

## Code availability

The Python source code for this project is publicly available at https://github.com/neurodatascience/ukbb-cca. The version of the code that produced results presented in the paper has been deposited to the Zenodo data repository^61^.
