# Supplementary material for: Replicability of multivariate brain-behaviour associations depends on clinical profile
Source: Commun Biol. 2026 Jun 25;9:1361. doi: 10.1038/s42003-026-10364-z (PMC13677668; doi:10.1038/s42003-026-10364-z)
Supplement: Supplementary file 2 [file 42003_2026_10364_MOESM2_ESM.pdf]

nature portfolio

Corresponding author(s): Michelle Wang, Jean-Baptiste Poline

Last updated by author(s): May 7, 2026

Reporting Summary

Nature Portfolio wishes to improve the reproducibility of the work that we publish. This form provides structure for consistency and transparency in reporting. For further information on Nature Portfolio policies, see our Editorial Policies and the Editorial Policy Checklist.

Statistics

For all statistical analyses, confirm that the following items are present in the figure legend, table legend, main text, or Methods section.

n/a	Confirmed	
☐	☒	The exact sample size (n) for each experimental group/condition, given as a discrete number and unit of measurement
☐	☒	A statement on whether measurements were taken from distinct samples or whether the same sample was measured repeatedly
☐	☒	The statistical test(s) used AND whether they are one- or two-sided<br>*Only common tests should be described solely by name; describe more complex techniques in the Methods section.*
☐	☒	A description of all covariates tested
☒	☐	A description of any assumptions or corrections, such as tests of normality and adjustment for multiple comparisons
☐	☒	A full description of the statistical parameters including central tendency (e.g. means) or other basic estimates (e.g. regression coefficient) AND variation (e.g. standard deviation) or associated estimates of uncertainty (e.g. confidence intervals)
☒	☐	For null hypothesis testing, the test statistic (e.g. F, t, r) with confidence intervals, effect sizes, degrees of freedom and P value noted<br>*Give P values as exact values whenever suitable.*
☒	☐	For Bayesian analysis, information on the choice of priors and Markov chain Monte Carlo settings
☒	☐	For hierarchical and complex designs, identification of the appropriate level for tests and full reporting of outcomes
☒	☐	Estimates of effect sizes (e.g. Cohen's d, Pearson's r), indicating how they were calculated

Our web collection on statistics for biologists contains articles on many of the points above.

Software and code

Policy information about availability of computer code

Data collection	We performed secondary data analysis; no additional data collection was done.
Data analysis	The Python source code for this project is publicly available at https://github.com/neurodatascience/ukbb-cca.

For manuscripts utilizing custom algorithms or software that are central to the research but not yet described in published literature, software must be made available to editors and reviewers. We strongly encourage code deposition in a community repository (e.g. GitHub). See the Nature Portfolio guidelines for submitting code & software for further information.

Data

Policy information about availability of data

All manuscripts must include a data availability statement. This statement should provide the following information, where applicable:

- Accession codes, unique identifiers, or web links for publicly available datasets
- A description of any restrictions on data availability
- For clinical datasets or third party data, please ensure that the statement adheres to our policy

Data used in this study can be obtained by applying for access to the UK Biobank database (https://www.ukbiobank.ac.uk/).
Source data for figures is available at https://github.com/neurodatascience/ukbb-cca and has been deposited to the Zenodo data repository at https://doi.org/10.5281/zenodo.20026935.

Research involving human participants, their data, or biological material

Policy information about studies with human participants or human data. See also policy information about sex, gender (identity/presentation), and sexual orientation and race, ethnicity and racism.

Reporting on sex and gender	Biological sex was used as a confounding variable in this analysis. The biological sex measure in the UK Biobank database is a mixture of the sex the National Health Service had recorded for the participant and self-reported sex. The gender measure was not available in the dataset.<br><br>This study did not perform sex-based analyses because there is no evidence in current literature that sex affects the reproducibility of brain-behaviour associations.
Reporting on race, ethnicity, or other socially relevant groupings	Self-reported ethnicity from the UK Biobank database was used as confounding variable in this analysis. The majority of UK Biobank participants (94.6%) is of white ethnicity, which reflects the national population in the UK around the time of recruitment.
Population characteristics	The UK Biobank is a large population study based in the United Kingdom.<br><br>The data sample used in this study consisted of 40,514 individuals, with a male-female split of 48.3% to 51.7%. The mean age was 64.2 with a standard deviation of 7.8. The vast majority of participants reported their ethnicity being White (96.8%); other participants reported being Mixed (0.5%), Asian/Asian British (1.1%), Black/Black British (0.7%), Chinese (0.3%), or none of the choices (0.5%).<br><br>This study further divided the sample described above into three cohorts: healthy participants (6676 samples), participants with past psychoactive substance use (4725 samples), and participants with hypertension (7768 samples).
Recruitment	The UK Biobank defines their recruitment strategy in Allen et al., 2024 (https://doi.org/10.1126/scitranslmed.adf4428): "Postal invitations were sent to 9.2 million individuals aged 40 to 69 who were registered with the UK's National Health Service (NHS) and lived within a short traveling time (typically about 25 miles) of 1 of 22 dedicated assessment centers. The choice of their location was determined by population density, ease of access, and potential to reach certain types of participants (e.g., ethnic minority groups and those living in more socioeconomically deprived areas). During 2006 to 2010, 502,000 participants were recruited (5.5% of those invited). Although the participation rate was low and those who joined the study were somewhat healthier and wealthier than the UK population across the same age range, the cohort includes large numbers of individuals across a broad spectrum of potential risk factors that vary from low to high exposure."
Ethics oversight	The UK Biobank study is approved by the North West Multi-centre Research Ethics Committee.

Note that full information on the approval of the study protocol must also be provided in the manuscript.

Field-specific reporting

Please select the one below that is the best fit for your research. If you are not sure, read the appropriate sections before making your selection.

☒ Life sciences ☐ Behavioural & social sciences ☐ Ecological, evolutionary & environmental sciences

For a reference copy of the document with all sections, see nature.com/documents/nr-reporting-summary-flat.pdf

Life sciences study design

All studies must disclose on these points even when the disclosure is negative.

Sample size	This study used the maximal number of available samples with magnetic resonance imaging (MRI) data from the UK Biobank at the time.
Data exclusions	Samples available in the precomputed derivatives have already passed quality control. Our exclusions of subjects were based on incomplete entries of the required variables.
Replication	A major part of our study design was performing extensive out-of-sample validations of the Canonical Correlation Analysis (CCA) model trained with different sizes and compositions of samples.
Randomization	Our work uses an observational design. The goal was to replicate when a modelled brain-behaviour relationship becomes stable within a general sample. Key variables were immutable characteristics of individuals; they were not assignable conditions. Individuals were randomly held out of the training sample and used to assess the replicability of the learned brain-behaviour associations.
Blinding	There was no group assignment to blind in our design.

Reporting for specific materials, systems and methods

We require information from authors about some types of materials, experimental systems and methods used in many studies. Here, indicate whether each material, system or method listed is relevant to your study. If you are not sure if a list item applies to your research, read the appropriate section before selecting a response.

Materials & experimental systems

n/a	Involved in the study
☒	☐ Antibodies
☒	☐ Eukaryotic cell lines
☒	☐ Palaeontology and archaeology
☒	☐ Animals and other organisms
☒	☐ Clinical data
☒	☐ Dual use research of concern
☒	☐ Plants

Methods

n/a	Involved in the study
☒	☐ ChIP-seq
☒	☐ Flow cytometry
☐	☒ MRI-based neuroimaging

Plants

Seed stocks

Report on the source of all seed stocks or other plant material used. If applicable, state the seed stock centre and catalogue number. If plant specimens were collected from the field, describe the collection location, date and sampling procedures.

Novel plant genotypes

Describe the methods by which all novel plant genotypes were produced. This includes those generated by transgenic approaches, gene editing, chemical/radiation-based mutagenesis and hybridization. For transgenic lines, describe the transformation method, the number of independent lines analyzed and the generation upon which experiments were performed. For gene-edited lines, describe the editor used, the endogenous sequence targeted for editing, the targeting guide RNA sequence (if applicable) and how the editor was applied.

Authentication

Describe any authentication procedures for each seed stock used or novel genotype generated. Describe any experiments used to assess the effect of a mutation and, where applicable, how potential secondary effects (e.g. second site T-DNA insertions, mosiacism, off-target gene editing) were examined.

Magnetic resonance imaging

Experimental design

Design type

The MRI data used diffusion MRI (dMRI), not functional, so task/resting is not a meaningful distinction. Data was distributed after preprocessing, with features computed and extracted into tabular form.

Design specifications

The MRI session acquisition was described by the UK Biobank. dMRI data does not have a task performed during acquisition: the only instructions were to stay still while looking at a fixation cross.

Behavioral performance measures

There was no behavioural task performed during dMRI data acquisition.

Acquisition

Imaging type(s)

dMRI, T1-weighted (T1w)

Field strength

3.0 Tesla

Sequence & imaging parameters

T1w: 3D MPRAGE acquisition with 1 mm isotropic resolution. Orientation Sagittal; Phase Enc. Dir. = A >> P; FoV Read=256 mm; FoV Phase=100.0 %; Slice Thickness=1.00 mm; TR=2000.0 ms; TE=2.01 ms; Averages=1.

Diffusion: Slices=72; Phase enc. dir. A >> P & P >> A; FoV Read=210 mm; FoV Phase=100.0%; Slice thickness=2.00 mm; TR=3600 ms; TE=92.00 ms; Multi-band accel. Factor=3

Area of acquisition

Whole brain

Diffusion MRI ☒ Used ☐ Not used

Parameters

Multi-shell (b=1000 and b=2000), with 50 directions per shell.

Preprocessing

Preprocessing software

The processed and extracted data provided by the UK Biobank were used. Detailed preprocessing procedure is described in Alfaro-Almagro et al. 2018 (https://doi.org/10.1016/j.neuroimage.2017.10.034). Briefly, their pipeline was composed of tools from FSL that handle the standard denoising and normalization.

Normalization

Most operations were performed in subject space. Normalization of the T1w image to the MNI template was estimated so the inverse transform could be applied to move standard space labels to subject space.

Normalization template

The MNI152NLin2009c template was used as a target, but ROIs were transformed from template to subject space for extraction.

Noise and artifact removal

Eddy currents, motion, scanner noise, and bias fields were all removed as part of processing.

Volume censoring

No volume censoring was performed as it is not a standard operation for dMRI processing.

Statistical modeling & inference

Model type and settings

CCA is a multivariate model that can be thought of as a correlation between two (or more) domains. If variables in a dataset can be separated by modality (e.g., brain and behaviour) then CCA finds linear combinations of variables in each modality such that these linear combinations (latent factors called "canonical axes") are maximally correlated with each other.

Effect(s) tested

The main results of an analysis involving CCA also include canonical correlations between brain and behavioural features and the canonical loadings of the brain and behaviour features on their respective axis. Canonical correlations are the correlations between each pair of canonical factors, and canonical loadings describe how features from the original data contribute to the canonical factors. Canonical loadings are obtained by correlating the original data matrices with the canonical factors.

Specify type of analysis: ☐ Whole brain ☒ ROI-based ☐ Both

Anatomical location(s)

A whole brain atlas extraction of the JHU DTI White Matter Atlas was used to create the ROI features used in the analysis.

Statistic type for inference

(See Eklund et al. 2016)

Neither voxel-wise or cluster-wise analysis was performed in this study.

Correction

No correction was done.

Models & analysis

n/a	Involved in the study
☒	☐ Functional and/or effective connectivity
☒	☐ Graph analysis
☐	☒ Multivariate modeling or predictive analysis

Multivariate modeling and predictive analysis

Inputs to the Canonical Correlation Analysis (CCA) models consisted of 162 dMRI weighted means measures and 43 cognitive test results (reaction time, numeric memory, fluid intelligence/reasoning, trail making, matrix pattern completion, tower rearranging, symbol digit substitution, prospective memory, and pairs matching). 18 demographic, physical and physiological variables were treated as confounds and regressed out of the input variables.

Principal Component Analysis (PCA) was used to reduce the dimension of the brain and behavioural variable sets to 20 principal components each. Canonical correlations for the Train and Test data were used to evaluate model fits.
