# Supplementary material for: Replicability of multivariate brain-behaviour associations depends on clinical profile
Source: Commun Biol. 2026 Jun 25;9:1361. doi: 10.1038/s42003-026-10364-z (PMC13677668; doi:10.1038/s42003-026-10364-z)
Supplement: Supplementary file 3 [file 42003_2026_10364_MOESM3_ESM.pdf]

nature portfolio

Peer Review File

Replicability of multivariate brain-behaviour associations depends on clinical profile

Corresponding Author: Ms Michelle Wang

This file contains all reviewer reports in order by version, followed by all author rebuttals in order by version.

Version 0:

Reviewer comments:

Reviewer #1

(Remarks to the Author)
Thank you for the opportunity to review this manuscript. the authors repeatedly fit CCA models across sample sizes and four cohorts (Full, Healthy, Psychoactive, Hypertension). using diffusion MRI and cognitive data from the UK Biobank (N=40,514). Test-set correlations improved with sample sizes and exceeded null expectations and loadings stabilised from around N≈500.
Strengths
(1) One of the key strengths of the paper is that it addresses the important question of how sample size influences the generalisability of canonical correlation analysis (CCA) – this is critical both interpreting existing literature and for future study desing.
(2) The paper is generally methodologically sound. The repeated train–test framework is clearly described, and the cohort comparison is straightforward
(3) The analysis extends beyond correlations to assess the stability of variable loadings, consistent with common interpretations of CCA in practice. Null (shuffled) models provide a baseline for assessing performance relative to chance.
(4) The authors provide their code and describe clearly the workflow
Areas for consideration
(1) There is a small inconsistency in the description of the cognitive measures. If I understood correctly, the results mention eight cognitive tests, whereas nine are listed in the methods.
(2) The paper would benefit from a more expanded and justified explanation for the rationale of confounding variables. For example, it appears that age and year of birth are excluded from the set of "primary demographic" confounds.
(3) The modelling pipeline relies on several fixed design choices (e.g., 20 principal components per domain, 20 canonical axes, and unregularised CCA). These decisions could affect stability and out-of-sample performance, especially at smaller sample sizes. So additional sensitivity analyses would help with interpreting the validity of the results.
(4) The interpretation of "significant" loadings would benefit from further clarification. The figures apply null-percentile masking, which makes is unclear as to how this relates to multiple testing across variables or how stable the sign and ordering of loadings are across repeats.
(5) Cohort definitions are broad (e.g., Psychoactive includes Z86.4 and any F10–F19 codes). While this not unreasonable it also complicates understanding of the effect of cohort composition as the cohorts appear rather heterogeneous. This is perhaps one of the weakest points of the paper and the authors may wish to downplay the importance of these findings.

Reviewer #2

(Remarks to the Author)
This is an interesting manuscript that builds on ongoing debates within the field regarding the minimum sample size needed to detect brain-behavior relationships that are reliable and reproducible. It seeks to address the claim that relative to large heterogeneous datasets such as ABCD, more homogenous samples, such as those composed of individuals who share a given diagnosis, may require smaller sample sizes to obtain replicable brain-behavior associations, particularly if methods used employ built-in cross-validation. These are issues that are already discussed in the literature in general, but there is limited empirical data and this study therefore addresses an important gap in the extant literature.

Specifically, the authors use canonical correlation analysis (CCA) with and without cross-validation across four different

‘cohorts’ of individuals from the UK Biobank: all participants, healthy participants, participants with substance-use, participants with hypertension. Within each cohort they run CCA in subsets of data across 30 different sample sizes and then test the reproducibility of these findings in a random split-half of the remaining individuals in the cohort.

Findings indicate that the minimum sample size required for reproducible brain-behavior associations differs as a function of cohort, with the smallest sample size required for the substance use cohort and the largest needed for the healthy cohort.

Across all cohorts, minimum required sample sizes hovered around 500, and CCA results for samples lower than ~200 were unstable. These findings algin with what most individuals who use CCA would probably have guessed however to my knowledge are one of the first if not the first empirical demonstration of this effect in a systematic manner. Similarly, as might also be expected, CCA without cross-validation in small samples was highly prone to over-fitting. Somewhat more surprisingly, cross-validation did not reduce over-fitting much for samples larger than 100 (though note that effect size inflation from train to test remains high in samples less than 1000). One limitation is that all analyses used diffusion data, therefore it is unclear how these findings might or might not also hold relevance for analysis of functional data.

In short, this is a nice contribution to the literature that will likely be of general interest to the neuroimaging community. A few questions and suggestions to further improve this submission are provided below.

Participants with psychoactive substance-use required the smallest sample sizes to obtain reproducible associations. Could the authors speculate as to why this might be? For example, might this relate to the effects of substance-use on white matter microstructures? Or a general effect of psychopathology?

More information on the types of substance-use should be included in the main text. There is also significant overlap between the psychoactive and hypertension samples. It will be important to test whether these participants (who presumably have the most white matter impairments) are driving effects. In other words, is it just that, for individuals with widespread white matter alterations, smaller sample sizes are needed OR is it that the variance that is being partitioned by the sub-grouping relates more to non-brain-derived IDPs? Attempting to address this would be of significant help to the field, as there are longstanding debates about whether heterogeneity of brain vs behavior contributes most to issues of reproducibility.

Reviewer #3

(Remarks to the Author)
This article provides a useful and massive data processing. The main message seems to be: focus the analysis on different categories (Psychoactive, Hypertensive, etc.) and, instead of thousands, you will need hundreds of cases to train your model. This is simple enough: simplify your analyses and build faster, more reliable trained models. However, this seems to go in the opposite direction of modern statistics: Borrowing Strength and Learning from the Experiences of Others. That does not mean that all data are equally informative. But you can have hierarchical models (typically Bayesian) on which you do not have complete pulling, but partial pulling. The authors mention packages, but do not show any modelling to be more incisive. I hope the authors send a revised manuscript that is more explicit about their modelling approach. Perhaps previous modelling has too much pulling, or the categories are so distinct that they ought to be individualized... But you have to convince your audience. On the other hand, this manuscript makes a very interesting reading.
Luis Pericchi

Version 1:

Reviewer comments:

Reviewer #1

(Remarks to the Author)
The manuscript has been substantially revised. There are no outstanding unaddressed concerns, the new analyses support the original conclusions, and the scope limitations acknowledged in the responses are reasonable and consistently reflected in the revised text.

Reviewer #2

(Remarks to the Author)
The authors have been responsive to the reviewer comments.

Reviewer #3

(Remarks to the Author)
I agree with the author’s paragraph:
“We acknowledge that this study could be extended by exploring other models (e.g. Bayesian, or with regularization, etc.), but it would then be very hard to give a simple guideline on parametrized models, and therefore we consider this out of the scope of this specific work”
However, I suggest that a comment is included in the final revision, about possible further methodological extensions in these directions.

Lluis Pericchi

Open Access This Peer Review File is licensed under a Creative Commons Attribution 4.0 International License, which permits use, sharing, adaptation, distribution and reproduction in any medium or format, as long as you give appropriate credit to the original author(s) and the source, provide a link to the Creative Commons license, and indicate if changes were made.
In cases where reviewers are anonymous, credit should be given to 'Anonymous Referee' and the source.
The images or other third party material in this Peer Review File are included in the article's Creative Commons license, unless indicated otherwise in a credit line to the material. If material is not included in the article's Creative Commons license and your intended use is not permitted by statutory regulation or exceeds the permitted use, you will need to obtain permission directly from the copyright holder.
To view a copy of this license, visit https://creativecommons.org/licenses/by/4.0/

Reviewer #1 (Remarks to the Author):

We thank the reviewer for their time.

1. **There is a small inconsistency in the description of the cognitive measures. If I understood correctly, the results mention eight cognitive tests, whereas nine are listed in the methods.**

   There was indeed an inconsistency. The correct number is nine cognitive tests. We corrected the Results section of the manuscript to reflect this:

   "Behavioural data consisted of key performance measures from nine cognitive function tests performed at the assessment centres."

2. **The paper would benefit from a more expanded and justified explanation for the rationale of confounding variables. For example, it appears that age and year of birth are excluded from the set of "primary demographic" confounds.**

   We have updated the relevant Methods subsection in the manuscript to refer to previous publications that informed our choice of confounding variables:

   "18 confounding variables were identified and regressed out of the Brain and Behavioural datasets. These measures were chosen based on methods described in previous work[1,2]. They consisted of the following:

   1. Primary demographics, including sex and assessment center but excluding age (Category 1001, except Field 21003 "Age when attended assessment centre" and Field 34 "Year of birth");
   2. Blood pressure (Category 100011, except for Field 96 "Time since interview start at which blood pressure screen(s) shown");
   3. Handedness (Field 1707);
   4. Pulse rate (Field 4194);
   5. Standing height (Field 50);
   6. Weight (Field 21002) and weight method (Field 21).

   Some demographic fields, such as sex, were not recorded during the first imaging visit; in these cases, the value at Instance 0 was used for all participants.

   Age-related data were not included as confound variables due to evidence that age may be more of an effect modifier than a confounder: brain-behavioural CCA associations are strongly linked with age, and including age as a confound reduces canonical correlations to near zero[2]."

3. **The modelling pipeline relies on several fixed design choices (e.g., 20 principal components per domain, 20 canonical axes, and unregularised CCA). These decisions could affect stability and out-of-sample performance, especially at smaller sample sizes. So additional sensitivity analyses would help with interpreting the validity of the results.**

Indeed, the fixed design choices we presented could have affected our results. We attempt to address these dimensions of analytic variability below.

Regarding the number of principal components (PCs), our original choice of 20 PCs for each domain was partly chosen so that the total number of input variables to the CCA model (40) would not exceed the smallest sample size used (50). These numbers corresponded to a percent variance explained of 92% for the behavioural domain and 71% for the brain domain. We have since rerun our analysis using a grid search of all combinations of 70%, 80% and 90% variance explained for each domain (i.e., 70% behavioural and 70% brain, 70% behavioural and 80% brain, etc.). We find that the number of PCs does not impact our main findings. We have updated the Results section with this new analysis and added two new figures in Supplementary Information:

"To test the robustness of these results, we conducted an additional sensitivity analysis (Supplementary Figure 3) where we varied the number of features used as input to the CCA model (see **Methods**). We compared the minimum sample size needed for each cohort to reach a mean r_{test} of 0.24 (corresponding to the r_{test} at sample size 487 for the Psychoactive cohort in Figure 2). The Psychoactive cohort consistently required the lowest sample size (between 487 and 907) compared to the Full (between 907 and 1116), Healthy (between 1372 and 2077), and Hypertension (between 907 and 1372) cohorts (Supplementary Figure 4)."

Regarding the canonical axes (CAs), we used 20 CAs (since that was the maximum possible with 20 PCs per domain), but only presented results on the first CA (CA1), which was the only one with non-null test correlation across all four cohorts (See Supplementary Figure 12). We would have obtained the exact same results for CA1 if we had chosen any number of CAs between 1 and 20 inclusive.

Regarding the use of regularization in the CCA model: indeed, adding regularization could change results, especially at smaller sample sizes. However, most brain-behavioural CCA studies – and notably the Marek *et al*. 2022 paper[3] to which this paper is partly a response – do not report using regularization, and so investigating the effect of regularization was outside the scope of this current study. We have updated the Discussion to mention that analytical choices like regularization of CCA may also impact the replicability of results:

"For example, collecting a more specialized set of features could lead to a sufficiently powered CCA with a much smaller sample. The same could be said about MRI modality, though a recent review of multivariate brain-behavioural relationship comparisons across

clinical conditions found that cortical morphology and white matter association tracts had an overall stronger impact on a brain-behaviour axis than resting state fMRI[4]. In parallel, analytical choices such as using regularized CCA[5] could impact the replicability of results as well. Such comparisons were beyond the scope of this study, but could form the basis of future work in this field."

4. **The interpretation of "significant" loadings would benefit from further clarification. The figures apply null-percentile masking, which makes it unclear as to how this relates to multiple testing across variables or how stable the sign and ordering of loadings are across repeats.**

   We used the term "significant" in a specific way, which could have caused misunderstanding. The null model loading percentiles were used as effect size thresholds to identify loadings that could be reasonably included in qualitative order comparison. We did not perform formal hypothesis testing. Regarding the stability of the loading signs and order, Supplementary Figures 8–11 show violin plots of the distribution of each loading at the highest sample size for each cohort, and Supplementary Figure 5 shows the standard deviation of every loading for all sample sizes. In general, the goal was to show that the set of strong loadings was conserved across sample sizes – the exact ordering of those loadings mattered less, as sampling variation could reasonably lead to some loadings to swap ranks if their true magnitudes were similar.

   We have reworded text in the Results and Discussion sections to replace "significant loadings" with "strongest loadings" or "loadings that pass null model thresholds".

5. **Cohort definitions are broad (e.g., Psychoactive includes Z86.4 and any F10–F19 codes). While this is not unreasonable it also complicates understanding of the effect of cohort composition as the cohorts appear rather heterogeneous. This is perhaps one of the weakest points of the paper and the authors may wish to downplay the importance of these findings.**

   Cohort definitions were determined based on available clinical information in the dataset, keeping in mind that the resulting cohorts needed to have reasonably large sample sizes. We agree that our cohort definitions could be considered broad in some regard, but they are still more precise than the general population sample (Full cohort). In fact, even with those arguably heterogeneous cohorts, we already found differences that support the idea that more targeted cohorts require smaller sample sizes to be replicable. We expect that, with even more precise cohorts, we would require even less samples to achieve replicability. We also looked at non-overlapping Psychoactive and Hypertension cohorts and found similar results, as shown in Supplementary Figure 13. This additional analysis is now mentioned in the Methods section:

   "The Psychoactive and Hypertension cohorts had 1989 participants in common – these did not seem to be driving the effects we found, as removing them from the analysis still

showed that the Psychoactive cohort needed less samples compared to the Healthy or Hypertension cohorts (Supplementary Figure 13)."

In addition, we have added a paragraph to the Discussion section to add nuance that cohort specification is not necessarily straightforward, and that clinical homogeneity might not always necessarily lead to stronger brain-behavioural associations.

"In general, although our results support the notion that targeted cohort selection can reduce the sample size requirements for multivariate brain-behaviour analyses, we acknowledge that this may not always be the case depending on the cohort. Cohort specification is a procedure that can span multiple dimensions of subject characteristics: disease itself is a multidimensional trait, and it typically interacts with demographic, socioeconomic, and genetic features. It is likely that not all dimensions would impact brain-behaviour associations in the same manner. Therefore, in some cases it is possible that what appears to be a more clinically homogeneous cohort shows in practice smaller brain-behaviour effect sizes."

Reviewer #2 (Remarks to the Author):

We thank the reviewer for their time.

1. **Participants with psychoactive substance-use required the smallest sample sizes to obtain reproducible associations. Could the authors speculate as to why this might be? For example, might this relate to the effects of substance-use on white matter microstructures? Or a general effect of psychopathology?**

   We agree this is an interesting idea, and after a brief search of the literature we made the following change to the manuscript to more completely address the question.

   “We found that using a targeted subset of participants with current or past psychoactive substance use resulted in higher replicability of brain-behaviour canonical correlations when compared to other participant cohorts at the same sample size. Psychoactive substance use is known to affect both cognitive function[6] and white matter microstructure[7]: a change in either domain could change the observed relationship in our cohort[4]. The exact cause of this effect is unclear from our current analysis, and deeper interpretation of this specific finding was beyond the scope of our study. However, sensitivity analyses suggest that the behavioural domain could be important, as the effect is less marked with fewer behavioural measures (Supplementary Figure 3), but more systematic analyses would be needed to draw concrete conclusions.”

2. **More information on the types of substance-use should be included in the main text.**

   We have named some examples of psychoactive substances in the Results section:

   “From the Full cohort of 40,514 participants, we created three smaller cohorts targeting subsets of healthy participants (Healthy cohort, 6676 samples), participants with past psychoactive substance (e.g., alcohol, opioids, cannabinoids, sedatives or hypnotics, cocaine) use (Psychoactive cohort, 4725 samples), and participants with hypertension (Hypertension cohort, 7768 samples) (see **Methods** for details).”

   We have added a link a relevant ICD-10 webpage https://icd.who.int/browse10/2019/en#/F10-F19 to the Cohort selection subsection of the Methods section:

   “The selection criteria consisted of the following:

   1. **Full**: all available participants (i.e., no filtering);
   2. **Healthy**: participants without any diagnosis code;
   3. **Psychoactive**: participants with code Z86.4 “Personal history of psychoactive substance abuse” or any code in the F10-F19 categories (“Mental and behavioural disorders due to psychoactive substance use”) (see https://icd.who.int/browse10/2019/en#/F10-F19 for more detailed information);
   4. **Hypertension**: participants with code I10 “Essential (primary) hypertension”.”

3. **There is also significant overlap between the psychoactive and hypertension samples. It will be important to test whether these participants (who presumably have the most white matter impairments) are driving effects. In other words, is it just that, for individuals with widespread white matter alterations, smaller sample sizes are needed OR is it that the variance that is being partitioned by the sub-grouping relates more to non-brain-derived IDPs?**

   We repeated our sample size analysis after removing overlapping participants between the Psychoactive and Hypertension cohorts. We also included as a separate cohort the overlapping subset of participants. We conclude that this subset of participants is not driving effects, though it is unclear how they interact in the context of the main analysis. Our results are shown in Supplementary Figure 13 and described in the "Cohort selection" subsection of the Methods:

   "The Psychoactive and Hypertension cohorts had 1989 participants in common – these did not seem to be driving the effects we found, as removing them from the analysis still showed that the Psychoactive cohort needed less samples compared to the Healthy or Hypertension cohorts (Supplementary Figure 13)."

   Regarding the question of domain contribution to the sample size requirement, we refer to our answer to point #2 above, in which we speculate that it is the behavioural variables that matter more, based on our findings in the grid search sensitivity analysis (Supplementary Figure 3).

Reviewer #3 (Remarks to the Author):

This article provides a useful and massive data processing. The main message seems to be: focus the analysis on different categories (Psychoactive, Hypertensive, etc.) and, instead of thousands, you will need hundreds of cases to train your model. This is simple enough: simplify your analyses and build faster, more reliable trained models. However, this seems to go in the opposite direction of modern statistics: Borrowing Strength and Learning from the Experiences of Others. That does not mean that all data are equally informative. But you can have hierarchical models (typically Bayesian) on which you do not have complete pulling, but partial pulling. The authors mention packages, but do not show any modelling to be more incisive. I hope the authors send a revised manuscript that is more explicit about their modelling approach. Perhaps previous modelling has too much pulling, or the categories are so distinct that they ought to be individualized... But you have to convince your audience. On the other hand, this manuscript makes a very interesting reading.
Luis Pericchi

We appreciate the reviewer's straight message and their positive feedback on the usefulness and interesting aspect of the manuscript. We understand that concepts from modern Bayesian statistics such as borrowing information partially from prior distributions would add to the work. The decision to select a specific simple model (CCA) was deliberate: in the brain-behaviour research field, this technique is very widely used (cf. numerous publications including Marek et al. 2022[3] upon which this work is partly based), and we specifically designed a study to provide the community information on the population size that are required for a solid interpretation of CCA results. Our manuscript contains a detailed description of the methods used (feature selection, confound removal, dimensionality reduction, CCA). We acknowledge that this study could be extended by exploring other models (e.g. Bayesian, or with regularization, etc.), but it would then be very hard to give a simple guideline on parametrized models, and therefore we consider this out of the scope of this specific work. We do explore the number of principal components in the behaviour and brain domains in the revised version and show the robustness of our study results with respect to this hyperparameter.

References

1. Smith, S. M. *et al.* A positive-negative mode of population covariation links brain connectivity, demographics and behavior. *Nat. Neurosci.* **18**, 1565–1567 (2015).
2. McPherson, B. C. & Pestilli, F. A single mode of population covariation associates brain networks structure and behavior and predicts individual subjects' age. *Commun. Biol.* **4**, 1–16 (2021).
3. Marek, S. *et al.* Reproducible brain-wide association studies require thousands of individuals. *Nature* **603**, 654–660 (2022).
4. Vieira, S. *et al.* Multivariate brain-behaviour associations in psychiatric disorders. *Transl. Psychiatry* **14**, 231 (2024).
5. Mihalik, A. *et al.* Canonical Correlation Analysis and Partial Least Squares for Identifying Brain–Behavior Associations: A Tutorial and a Comparative Study. *Biol. Psychiatry Cogn. Neurosci. Neuroimaging* **7**, 1055–1067 (2022).
6. Ramey, T. & Regier, P. S. Cognitive Impairment in Substance Use Disorders. *CNS Spectr.* **24**, 102–113 (2019).
7. Hampton, W. H., Hanik, I. M. & Olson, I. R. Substance Abuse and White Matter: Findings, Limitations, and Future of Diffusion Tensor Imaging Research. *Drug Alcohol Depend.* **197**, 288–298 (2019).

Reviewer's comments

I agree with the author's paragraph:
"We acknowledge that this study could be extended by exploring other models (e.g. Bayesian, or with regularization, etc.), but it would then be very hard to give a simple guideline on parametrized models, and therefore we consider this out of the scope of this specific work"
However, I suggest that a comment is included in the final revision, about possible further methodological extensions in these directions.

Authors reply

We have added the following to the Discussion:

Our main findings are consistent with the claim that lower sample sizes may be sufficient when effect sizes are expected to be larger due to study design[1–5]. It should be noted that our study only focused on varying cohort composition, while other factors such as feature selection likely also impact brain-behaviour associations. For example, collecting a more specialized set of features could lead to a sufficiently powered CCA with a much smaller sample. The same could be said about MRI modality, though a recent review of multivariate brain-behaviour relationship comparisons across clinical conditions found that cortical morphology and white matter association tracts had an overall stronger impact on a brain-behaviour axis than resting state fMRI[6]. In parallel, analytical choices such as using regularized CCA[7] or other models (e.g., Bayesian methods) could impact the replicability of results as well. Such comparisons were beyond the scope of this study, but could form the basis of future work in this field.

1. Gratton, C., Nelson, S. M. & Gordon, E. M. Brain-behavior correlations: Two paths toward reliability. *Neuron* **110**, 1446–1449 (2022).
2. Libedinsky, I. *et al.* Reproducibility of neuroimaging studies of brain disorders with hundreds -not thousands- of participants. 2022.07.05.498443 Preprint at https://doi.org/10.1101/2022.07.05.498443 (2022).
3. Rosenberg, M. D. & Finn, E. S. How to establish robust brain–behavior relationships without thousands of individuals. *Nat. Neurosci.* **25**, 835–837 (2022).
4. Kang, K. *et al.* Study design features increase replicability in brain-wide association studies. *Nature* **636**, 719–727 (2024).
5. DeYoung, C. G. *et al.* Beyond Increasing Sample Sizes: Optimizing Effect Sizes in Neuroimaging Research on Individual Differences. *J. Cogn. Neurosci.* **37**, 1023–1034 (2025).

6. Vieira, S. *et al.* Multivariate brain-behaviour associations in psychiatric disorders. *Transl. Psychiatry* **14**, 231 (2024).

7. Mihalik, A. *et al.* Canonical Correlation Analysis and Partial Least Squares for Identifying Brain–Behavior Associations: A Tutorial and a Comparative Study. *Biol. Psychiatry Cogn. Neurosci. Neuroimaging* **7**, 1055–1067 (2022).
